# Corns

**Published:** 1871-04

**Authors:** 


### CORNS.

The leaf of the ground ivy, bruised and applied to a hard corn, is said to be a speedy cure for the common corns on our toes; it ought to be known that the fresh gizzard of a dead goose will do the same thing, if renewed frequently. We certainly know that an oyster's heart will be equally efficient; in fact, there are so many unfailing cures for corns, that it is a wonder there is a single one left in the world. Instead of burdening the memory with a variety of intricate and impossible preparations, it is easier to remember the principles involved. First, remove the pressure or friction of the shoe which occasioned the corn. Second, keep it moist long enough, and it will drop out of itself, or can be easily picked out with the finger-nail; and if it ever comes again, repeat the process. Lockjaw has frequently followed cutting hard corns; at other times, convulsions and death. This risk ought never to be run as long as a bit of cotton saturated with water, or sweet oil, or, better still, glycerine, which is the essential element of sweet oil, is a safe, certain, and efficient cure for hard corns, if kept constantly applied for a day or two, and no shoe is worn.

